# Analysis of eligibility criteria in Alzheimer’s and related dementias clinical trials

**DOI:** 10.1038/s41598-024-65767-x

**Published:** 2024-07-01

**Authors:** Alexandra K. Mitchell, Rebecca Ehrenkranz, Sanne Franzen, Sae H. Han, Mujaahida Shakur, Melissa McGowan, Holly A. Massett

**Affiliations:** 1 Kelly Government, Kelly Services, Inc., Rockville, MD 20852 USA; 2 Department of Neurology, Erasmus MC University Medical Center, Rotterdam, The Netherlands; 3 Division of Extramural Activities, National Institute on Aging, National Institutes of Health, 7201 Wisconsin Ave., Ste 2S-603, Bethesda, MD 20814 USA

**Keywords:** Alzheimer disease, Dementia, Eligibility criteria, Patient selection, Clinical trials, Diversity, Equity, Inclusion, Health status disparities, Recruitment, Cognitive ageing, Alzheimer's disease

## Abstract

Overly restrictive clinical trial eligibility criteria can reduce generalizability, slow enrollment, and disproportionately exclude historically underrepresented populations. The eligibility criteria for 196 Alzheimer’s Disease and Related Dementias (AD/ADRD) trials funded by the National Institute on Aging were analyzed to identify common criteria and their potential to disproportionately exclude participants by race/ethnicity. The trials were categorized by type (48 Phase I/II pharmacological, 7 Phase III/IV pharmacological, 128 non-pharmacological, 7 diagnostic, and 6 neuropsychiatric) and target population (51 AD/ADRD, 58 Mild Cognitive Impairment, 25 at-risk, and 62 cognitively normal). Eligibility criteria were coded into the following categories: Medical, Neurologic, Psychiatric, and Procedural. A literature search was conducted to describe the prevalence of disparities for eligibility criteria for African Americans/Black (AA/B), Hispanic/Latino (H/L), American Indian/Alaska Native (AI/AN) and Native Hawaiian/Pacific Islander (NH/PI) populations. The trials had a median of 15 criteria. The most frequent criterion were age cutoffs (87% of trials), specified neurologic (65%), and psychiatric disorders (61%). Underrepresented groups could be disproportionately excluded by 16 eligibility categories; 42% of trials specified English-speakers only in their criteria. Most trials (82%) contain poorly operationalized criteria (i.e., criteria not well defined that can have multiple interpretations/means of implementation) and criteria that may reduce racial/ethnic enrollment diversity.

## Introduction

The number of older adults living with Alzheimer’s Disease and Related Dementias (AD/ADRD) in the United States is projected to increase to over 13 million by 2060^1^. This increase will be driven by the rise among African American/Black (AA/B) and Hispanic/Latino (H/L) populations, which have up to double the risk of developing AD/ADRD as non-Hispanic Whites^2^. Clinical trials are critical to develop new prevention and treatment modalities for AD/ADRD, as there are no curative AD/ADRD treatments. Currently, many AD/ADRD clinical trials fail to recruit representative samples, particularly from the populations most burdened by AD/ADRD^3–5^. According to a recent meta-analysis, only 11% of AD/ADRD trial participants are from an underrepresented racial/ethnic background^6^. One barrier impeding inclusion of racially and ethnically diverse participants in AD/ADRD clinical trials is the use of overly stringent eligibility criteria^7–9^. This paper presents findings from a content analysis of all eligibility criteria listed for U.S. federally funded AD/ADRD clinical trials active in the last five years to identify the most common criteria present and their potential to affect the inclusion of traditionally underrepresented populations.

Eligibility criteria are an integral part of clinical research and, when well-designed, help ensure that trial participants appropriately reflect the target population, reduce safety risks, and limit variability among participants^7,8^. However, overly restrictive trial eligibility criteria may reduce diverse enrollment without improving participant safety, thereby limiting generalizability^10,11^. A 2022 report by the National Academies of Sciences, Engineering, and Medicine (NASEM) highlighted eligibility criteria as a key barrier to equitable participation in clinical trials^12^. This is especially salient for AD/ADRD trials, as overly stringent criteria are particularly likely to exclude participants from historically underrepresented groups who may experience higher rates of dementia-related risk factors^2,4,7,13,14^.

New guidance has been developed to address the concerns surrounding clinical trial eligibility criteria. The NASEM committee suggested carefully designing and intentionally applying eligibility criteria to ensure they are specific to the trial objective and promoting education for researchers about the benefits of using less restrictive eligibility criteria^12^. In 2020, the Food and Drug Administration (FDA) issued formal guidance for developing eligibility criteria to increase enrollment of underrepresented clinical trials^15^. The FDA recommends: (1) reviewing criteria on a trial-by-trial basis to ensure they are representative of the population for the intended drug and reflect the clinically relevant populations; (2) avoiding transferring restrictions from Phase 2 to Phase 3 protocols; and, (3) considering trial designs that broaden enrollment (e.g., adaptive design)^15^.

The U.S. National Institute on Aging (NIA) is the primary federal institute overseeing the conduct of AD/ADRD clinical research and currently oversees more than 400 active AD/ADRD clinical trials. To date, there is no assessment of the eligibility criteria used within federally funded AD/ADRD clinical trials and their potential to affect inclusion of diverse participants. Understanding the types and frequency of eligibility criteria included within NIA’s portfolio of AD/ADRD trials will provide the first step to addressing the recommendations put forth by the FDA and NASEM to ensure that trial eligibility criteria reduce risks and maximize generalizability. This paper is the first to conduct and report on a meta-scientific study to analyze eligibility criteria in federally funded AD/ADRD trials. We conducted a content analysis of NIA-funded, AD/ADRD trials active between January 2018 through January 2023 (N = 196 trials) to (1) determine the most common eligibility criteria for NIA-funded AD/ADRD clinical trials, by trial type and target population; and (2) evaluate if widely used eligibility criteria have the potential to disproportionately exclude historically underrepresented populations in NIA-funded AD/ADRD clinical trials. The focus of this paper are federally funded interventional trials recruiting participants in the United States from along the AD/ADRD continuum of disease.

## Methods

### Clinical trial selection for inclusion in this analysis

All active NIA-funded AD/ADRD trials with project start dates between January 1, 2018 and January 1, 2023 were evaluated for inclusion in this content analysis (N = 420) and were identified using NIA’s Clinical Research Operations and Management System (CROMS, https://croms.nia.nih.gov/) platform. CROMS is an internal database that tracks all NIA-funded clinical trials and assists NIA in the management of its clinical trials’ enrollment data and study-specific information, including their eligibility criteria. For this analysis, trials were excluded if they included foreign sites or were not recruiting human subjects (e.g., secondary analysis) (n = 54). Trials designated by NIA as ‘Care and Caregiving’, where the intervention target population comprised of caregivers rather than those along the AD/ADRD continuum of disease, also were excluded (n = 170). A total of 196 trials were included in the final analysis. This study is not human subjects research.

These 196 selected trials were categorized by trial type using the NIA categorical framework^16^ (pharmacological, non-pharmacological, diagnostic tools, and treatment for neuropsychiatric symptoms) and by target population. The target population classifications were determined based on the continuum of cognitive status as defined by each trial: preclinical cognitively normal (normal baseline cognitive abilities), preclinical at-risk (normal baseline cognitive abilities with genetic predisposition or family history of AD/ADRD), MCI, and dementia stages (AD/ADRD)^17^. Criteria for an MCI diagnosis include changes in cognitive abilities and impairment in one or more cognitive domains, but no significant impairment in daily functioning^17^. An AD/ADRD diagnosis occurs when cognitive impairment and memory loss lead to significant impairment in social and occupational functioning^17^.

### Codebook development of eligibility criteria

A codebook was developed for this content analysis following established methods described in Krippendorff et al.^16^. Thirty percent of the eligible trials were randomly selected and reviewed to develop a codebook that represented eligibility criteria found within the NIA-funded trials; 128 mutually exclusive thematic categories were identified for the codebook. These 128 individual criteria were further grouped into one of five overarching mutually exclusive categories: Neurologic, Psychiatric, Medical, Procedural, and Demographic. The Neurologic category contained all non-psychiatric central nervous system conditions (e.g., ‘stroke’ and ‘traumatic brain injury’), the Psychiatric category contained all psychological diagnoses (e.g. ‘schizophrenia’ and ‘bipolar disorder’), and the Medical category contained all non-neurological and non-psychiatric conditions (e.g., ‘hypertension’ and ‘osteoporosis’). The Procedural category contained criteria related to participating in the trial itself (e.g. ‘transportation’ and ‘unwilling to be randomized’) and the Demographic category contained criteria related to participants’ demographic status (e.g., ‘gender’ and ‘race/ethnicity’).

### Coding procedures for eligibility criteria and examples

We extracted each trial’s respective list of inclusion and exclusion eligibility criteria from CROMS. The eligibility criteria in CROMS are entered by NIA-grantees at study commencement. For this analysis, eligibility criteria were coded as exclusions in the following manner: each listed exclusion criterion was coded as either a ‘1’ if present, or a ‘2’ if the criterion was present, but listed as an example (e.g., if a trial’s exclusion criteria stated ‘cognitive impairment such as traumatic brain injury,’ ‘cognitive impairment’ would be coded as a ‘1’ and ‘traumatic brain injury’ would be coded as ‘2’). Inclusion criteria were reverse coded as exclusions (e.g. ‘eligible to participate if at least age 65’ was reframed as ‘ineligible to participate if under age 65’ and that trial would be coded as ‘1’ in the age restriction column with an age minimum of 65 recorded in the age minimum column). If an inclusion criterion was reverse coded and resulted in the duplication of an exclusion criterion for that same trial, the criterion was only counted once. All criteria are henceforth referred to as “exclusion criteria”.

Because this content analysis was focused on AD/ADRD trials, exclusion criteria that directly determined the cognitive status of the population of interest were not coded. Thus, the ‘dementia/cognitive impairment’ category was included in the codebook to cover criteria such as impairment secondary to brain injury. For example, if the trial population was cognitively normal people, and thus excluded those with MCI or AD/ADRD, ‘dementia/cognitive impairment’ would not have been coded as an exclusion criterion because that criterion was used to define the target population.

### Coding procedures for poorly operationalized exclusion criteria and examples

During the coding process, several criteria across medical, neurologic, and psychiatric domains were nebulous and did not fall clearly into a specified category without subjective interpretation (e.g., ‘other significant health problems’ and ‘active major neurologic disorders’). To thematically document these loosely defined criteria, the codebook included the following eight mutually exclusive categories for poorly operationalized criteria: ‘unspecified medical condition,’ ‘unspecified neurologic condition,’ ‘unspecified psychiatric condition,’ ‘ruled out by exam,’ ‘abnormal neuroimaging,’ ‘cognitive impairment accompanying other illness or condition,’ ‘attitudinal or behavioral barrier,’ and ‘investigator/authority/supervisor's discretion’. These poorly operationalized categories followed the same rules described above if they were part of a list of criteria that included examples (e.g., ‘serious unspecified medical condition, such as cancer or diabetes’ would result in a code of ‘1’ for ‘unspecified medical condition’ and a code of ‘2’ for ‘diabetes’ and ‘cancer’).

### Coding procedures for language requirement

‘Language’ was coded as an exclusion category if any language restriction was listed among a trial’s exclusion criteria. Given the high relevance of language specificity on the inclusion of diverse participants into trials, a supplemental review of trials’ grant applications was conducted for those trials where language was not listed as an exclusion criterion. If a language requirement was specified in the grant application but not in the exclusion criteria list, it was noted and coded separately in order to conduct a secondary analysis of language restrictions among the trials.

### Sub-coding

Three of the codes in the codebook were further sub-coded to provide additional elaboration. Criteria that were coded as ‘cognitive impairment’ were subsequently assigned a sub-code indicating whether the impairment was ‘mild cognitive impairment,’ ‘mild/moderate ADRD’ or ‘severe dementia’. When a criterion was coded as ‘Language’, a sub-code indicated whether the requirement was ‘English only,’ ‘Spanish and/or English,’ ‘Other language and/or English,’ or ‘non-English’. Age requirements for trial participation were sub-coded to reflect the age ranges, if noted.

### Interrater reliability

Two authors (A.M. and R.E.) independently coded the 196 trials’ exclusion criteria across 128 total specific exclusion criteria. Ten percent of the trials were randomly selected to be double-coded; using Cohen’s κ, an interrater reliability of 0.9 was determined, representing strong interrater reliability^18^. Differences in coding were reviewed and resolved by consensus; clarifications were made to the codebook and data dictionary if needed. If changes to the codebook or data dictionary were made, trials were re-coded to ensure standardization.

### Health disparities documentation

A review of recent literature (2010 or later) was conducted to identify and document health disparities reported for AA/B, H/L, AI/AN, and NH/PI populations to identify exclusion criteria with the potential to disproportionately exclude persons who are traditionally underrepresented in AD/ADRD trials. Only systematic literature reviews, meta-analyses, and U.S. surveillance reports were included in the literature search to ensure that only consistently demonstrated and nationally representative findings were included. Exclusion criteria from the codebook, along with terms that are associated with prevalence data and ethno-racial disparities (e.g., ‘health disparity’) were searched using PubMed and Google Scholar. Health disparity data from included papers were extracted into an Excel spreadsheet. The following data from each relevant manuscript were extracted: study objective, study population, study design, and results (including effect sizes) for each ethnic/racial group. The number of trials that included criteria documented in the literature as disproportionately affecting underrepresented populations were counted, and the percent of trials that could potentially exclude these populations was calculated. Because this content analysis was focused on AD/ADRD trials and the target populations fall along the continuum of the AD/ADRD symptom spectrum, exclusion criteria that directly determined the cognitive status of the population of interest were not coded. Thus, while dementia/cognitive impairment disproportionately affects minoritized populations, this category was excluded from this health disparities analysis.

### Statistical analysis

Counts and percentages were calculated for each exclusion criterion across all trials. The average number, mean, median, minimum/maximum, and interquartile range of exclusions per trial were calculated by trial type and target population. Unadjusted ANOVA tests were run to assess differences in the average number of exclusion criteria by trial type and target population with pooled standard deviations and Bonferroni correction. Number, percentage, and rank of trials with poorly operationalized criteria were calculated. For all criteria with literature documenting disparate burden on minoritized populations, the total number and percentage of trials with that criterion were calculated. R version 4.2.2 was used to conduct ANOVA analyses and to create figures.

## Results

### Exclusion criteria by trial type

Table 1 provides a description of the trials included in the analysis. The majority of the 196 trials were non-pharmacological (n = 128; 65%), followed by pharmacological (n = 55; 28%). Of the 55 pharmacological trials, 48 were Phase I/Phase II trials. Only a relatively small number of trials focused on neuropsychiatric symptoms (n = 6) or diagnostic tools (n = 7). The trials aimed to enroll participants across the continuum of cognitive status (preclinical through AD/ADRD), though fewer trials focused on at-risk populations.

**Table 1:** Quantitative description of exclusion criteria among national institute on aging clinical trials (N = 196).

	Trial type	Total # of trials (N)	Average # of exclusion criteria/trial	Median # exclusion criteria/trial	Range	Interquartile range: quartile 1–3
	All trials	196	17.5	15	1–54	7 (11–18)
Trial type	Pharmacological	55	26.3	26	2–54	20 (15–35)
	*Phase I/II*	*48*	*27.4*	*28.5*	*2–54*	
	*Phase III/IV*	*7*	*18.4*	*18*	*8–26*	
	Non-pharmacological	128	14.2	12.5	1–38	6 (10–16)
	Diagnostic tools	7	17.1	15	9–28	4.5 (13.5–18)
	Treatment for NPS	6	9.3	9	4–14	9 (4–13)
Target population	Cognitively normal	62	15.8	14	1–36	13 (11–24)
	At-risk	25	19.3	16	6–48	16.5 (10.5–27)
	MCI	58	15.5	14	3–38	11.5 (10.5–22)
	AD/ADRD	51	21.1	20	2–54	21.5 (10.5–32)

Significant values are in italics.

*NPS* Neuropsychiatric symptoms, *Cognitively normal* normal baseline cognitive abilities, *At-risk* cognitively normal with genetic predisposition or family history of dementia, *MCI* Mild cognitive impairment, *AD/ADRD* Alzheimer's disease and related dementias.

**Figure 1:**
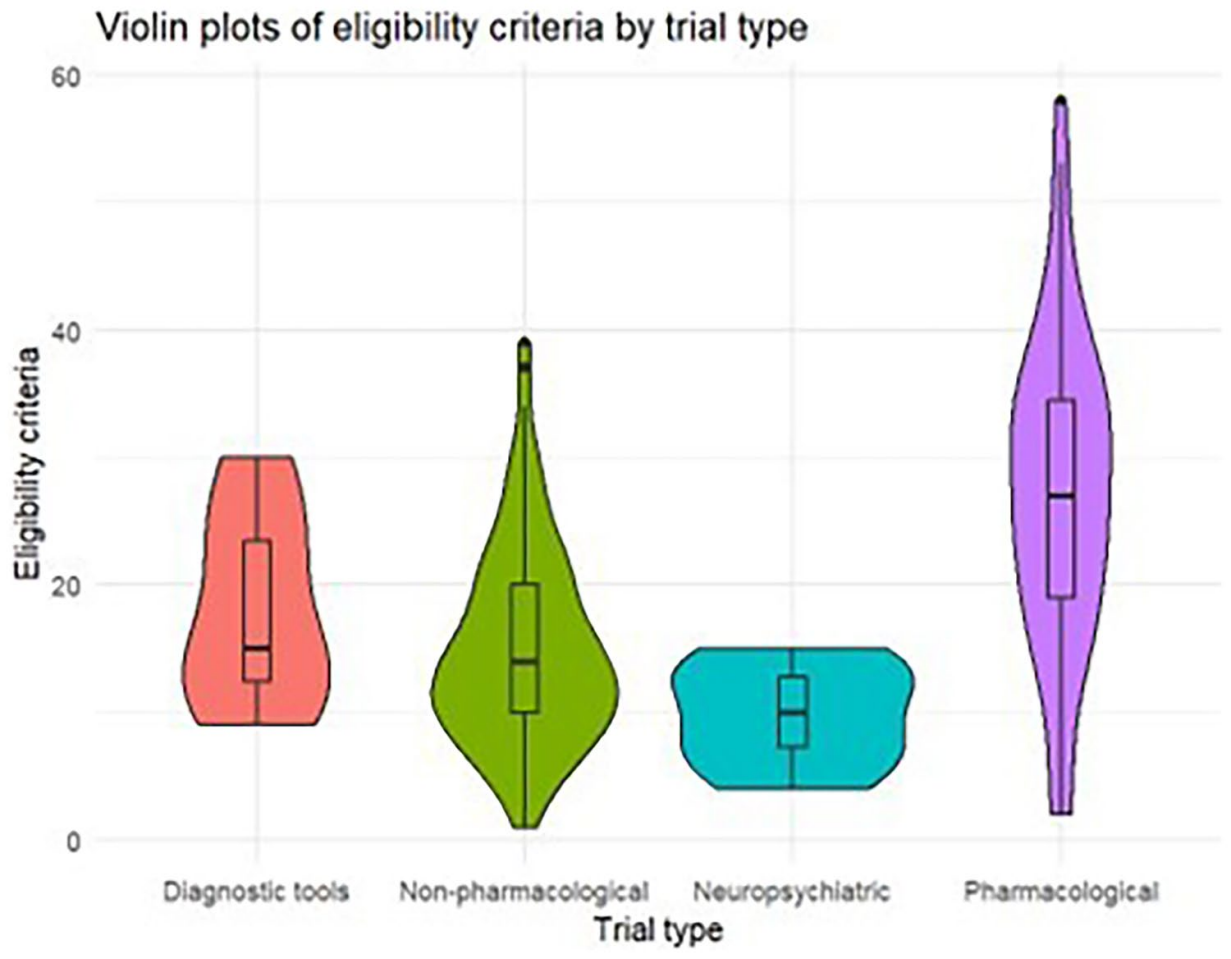
Plots of the number of exclusion criteria by trial type and target population.

A total of 3617 exclusion criteria were identified across the 196 clinical trials with a median of 15 exclusion criteria per trial (average = 17.5, range = 1–54) (Table 1). Figure 1 shows the distribution of exclusion criteria using violin plots (i.e., summary statistics with probability density). Phase I/II pharmacologic trials had the highest median (28.5) number of exclusion criteria relative to all other trial types, as well as the greatest number of exclusion criteria. By trial population, trials intervening on cognitively normal and MCI participants had lower median exclusion criteria (14) and were more concentrated around the median than trials intervening on at-risk or AD/ADRD participants (16). Trials enrolling those with AD/ADRD had the highest overall and average number of exclusion criteria and were more dispersed (i.e., narrower throughout).

One-way ANOVA revealed differences in the average number of exclusion criteria across the four trial types (F(3, 192) = 24.7, p < 0.001) (Supplemental Table 1). On average, pharmacological studies had more exclusion criteria than non-pharmacological studies (p < 0.001), while studies focused on treatment for NPS had significantly fewer exclusions per trial than non-pharmacological studies (p < 0.0001) (Supplemental Table 1). One-way ANOVA also revealed significant differences in the average number of exclusion criteria across target populations (F(3, 192) = 3.8, p = 0.01) (Supplemental Table 2): AD/ADRD-focused trials had significantly more exclusion criteria than trials in cognitively normal participants (p = 0.03) and MCI studies (p = 0.03) (Supplemental Table 2).

### Exclusion criteria by overall rank, number, and percent

Table 2 provides the 20 most frequent exclusion criteria identified in the analysis; these were identified in at least one-third of all trials. The top three most common exclusion criteria were age (87% of trials), specified neurologic disorders (65% of trials) and specified psychiatric disorders (61% of trials). Polypharmacy and substance use were used as exclusion criteria in at least half of all trials (54% and 50%, respectively). Of the top 20 most common exclusion criteria, ‘medical’ was the general category most frequently represented, with 10 of the top 20 criteria within the medical category.

**Table 2:** Rank, frequency, and percentage of exclusion criteria.

General category	Specific exclusion categories	Rank	Total # and % of trials with condition as criterion
Procedural/demographic	Age	1	171 (87%)
Neurological	Specified neurological disorders (including ALS, stroke, Lewy body, etc.)	2	128 (65%)
Psychiatric	Specified psychiatric (including depression, bipolar, OCD, etc.)	3	119 (61%)
Neurological	Unspecified neurological disorders	4	115 (59%)
Medical	Polypharmacy/concomitant medication	5	105 (54%)
Medical	Substance use/dependence	6	98 (50%)
Medical	Unspecified significant/severe medical condition	7	97 (49%)
Psychiatric	Unspecified psychiatric disorder	8	96 (49%)
Procedural/demographic	Language	9	93 (47%)
Cognitive	Dementia/cognitive impairment	10	92 (47%)
Medical	Cardiovascular/Congestive Heart Failure	11	88 (45%)
Medical	Visual impairment	12	82 (42%)
Procedural/demographic	Informed consent	13	77 (39%)
Medical	Cancer/malignancy/cancer therapeutics	14	71 (36%)
Medical	Hearing impairment	15	68 (35%)
Procedural/demographic	Association with another clinical trial	16	68 (35%)
Procedural/demographic	Contraindication to neuroimaging	17	67 (34%)
Medical	Disability/mobility impairment	18	63 (32%)
Medical	Pregnancy/pre-menopausal	19	59 (30%)
Medical	Contraindication to treatment/testing procedures	20	59 (30%)

Cognitive status was included in this table as a general category.

### Age-related exclusion

As noted above, age is the most common exclusion criterion identified among the NIA-funded trials, with nearly 90% of trials (n = 171) having an age requirement as part of their exclusion criteria. Of these trials, 100% listed an age minimum and 59% (n = 101) included an age maximum. The median minimum age was 55 with a range of 18–75 years old, and the median maximum age was 80 with a range of 35–90 years old. The highest age specified as the maximum eligible age was 90. The lowest average maximum age was 71.3 in trials of cognitively normal participants, and the highest average maximum age was 86.0 in trials of AD/ADRD participants. There were statistically significant differences in the average maximum age by continuum of disease (F(4,191) = 3.0, p = 0.02).

### Poorly operationalized exclusion criteria

Nearly half of the trials (46%) had at least three poorly operationalized exclusion criteria that are not well defined and can have multiple means of implementation. Eight total criteria were deemed poorly operationalized. Table 3 represents the counts, prevalence, and rank of these criteria across all included trials. Three of the eight criteria ranked in the top 10 list. ‘Unspecified neurologic’ conditions were listed in 58.7% of all trials and ranked as the 4th most common overall exclusion criterion. ‘Unspecified medical’ and ‘unspecified psychiatric’ conditions were each present in about half of all trials (49.5% and 49%, respectively) and ranked as the 7th and 8th most common exclusion criteria, respectively. Forty-five percent of trials (n = 89) had at least three poorly operationalized criteria listed, and 13% (n = 25) had five or more poorly operationalized exclusion criteria.

**Table 3:** Count, percent and rank of the exclusion criterion that are poorly operationalized.

Poorly operationalized exclusion criterion	N and % of total trials (N = 196) with criterion	Rank among total trials
Unspecified neurologic	115 (58.7)	4
Unspecified medical	97 (49.5)	7
Unspecified psychiatric	96 (49.0)	8
Investigator's discretion	53 (27.0)	23
Ruled out by exam	40 (20.4)	29
Abnormal brain test	17 (8.7)	45
Cognitive impairment accompanying other illness or condition	13 (6.6)	53
Attitudinal or behavioral barrier	13 (6.6)	54

Unspecified criteria represent criteria that were loosely defined in the original exclusion criteria, either with or without examples of the criterion (e.g., 'serious unspecified medical condition, such as cancer or diabetes') and without example (e.g., 'serious unspecified medical condition'). See methods for further description.

### Language exclusion criteria

Just under 50% of all trials specified a language as a restriction in the exclusion criteria (n = 93, 47%; ranked 9th of the 80 categories). Of these trials, 88% (n = 82) were available only to English-proficient participants, nine trials were available to English- and/or Spanish-proficient participants only, one trial was available to English- and/or Chinese-proficient participants only, and one trial required Spanish as the participant’s primary language. The remaining 103 trials (53%) did not specify language in their exclusion criteria. A review of these trials’ grant applications revealed that 41% (n = 42) of them did have a language requirement in their application though not listed in their exclusion criteria: 21 trials were available only to English-proficient participants; 13 trials were available to English- and/or Spanish-proficient participants; and one trial each was available to English-, Spanish-, and/or French-proficient participants, English-, Spanish-, Portuguese-, French-, Chinese-, Thai-, Japanese-, and/or Russian-proficient participants, and Native Hawaiian-proficient participant. The remaining 61 trials of the 196 (31%) made no reference or specification of language within their grant application or exclusion criteria, so it is unknown as to a language requirement restriction. As demonstrated in Table 4, if the additional 42 trials with language restrictions in their grant application had listed ‘language’ in their trials’ exclusion criteria, ‘language’ would be the second most common exclusion category within the 196 NIA-funded, AD/ADRD trials (n = 135 trials; 69%), with 53% (n = 103) of all trials specifying ‘English only’ and 11% (n = 22) specifying ‘English and/or Spanish’ proficiency.

**Table 4:** Language-based restrictions found in exclusion, protocol, and informed consent documents.

Source	Total # (%) of trials that specify language restriction	Total # (%) of trials that specify English-only	Total # (%) of trials that specify English- and/or Spanish-only
Exclusion criteria (n = 196)	93 (47)	82 (42)	9 (5)
Grant application (n = 103)	42 (41)	21 (20)	13 (13)
Total (n = 196)	135 (69)	103 (53)	25 (13)

31% of all trials (n = 61) did not specify a language restriction in the exclusion criteria or the grant application.

### Exclusion criteria with greater impact on diverse populations

Of the 128 exclusion categories, there were 16 criteria for which a systematic review or meta-analysis was found that described disproportionate effects of that criterion on the AA/B, H/L, AI/AN, or NH/PI populations (see Table 5). Given that many of the exclusion criteria are uncommon or affect only a relatively small percentage of the population (e.g., amyotrophic lateral sclerosis), available literature meeting our criteria was not found for such conditions. Literature was most commonly found for AA/B populations (literature found for 14 of the 16 criteria), followed by H/L (10 criteria), AI/AN (5 criteria), and NH/PI (4 criteria). No systematic reviews or meta-analyses were found for the conditions evaluated that documented excess incidence, prevalence, or burden among Asian populations.

**Table 5:** Count and frequency of exclusion criteria that may disproportionately reduce diverse enrollment.

Overarching category	Exclusion categories	Total # (%) trials with condition as criterion	Criteria affecting African American/Black participants	Criteria affecting Hispanic/Latino participants	Criteria affecting American Indian/Alaska Native participants	Criteria affecting Native Hawaiian/Pacific Islander participants
Medical	Substance use^50^	98 (50)	AA women had a higher rate of binge drinking relative to NHW women (10% vs 6%)		AI/AN had 43.4% (SE: 2.81) lifetime prevalence of alcohol use disorder compared to NHW and 1.8 (1.40–2.31) times the odds of severe alcohol use disorder compared to NHW	
	Renal^51^	56 (28.6)	HR for chronic kidney disease progression in AA/B ranges from 1.16 [1.09–2.62] to 4.00 [2.99–5.35]			
	Mobility impairment^52^	52 (26.5)	AA/B had greater difficulty walking than NHW (adjusted B = 0.41, CI 0.28–0.54)	H/L had greater difficulty walking than NHW (adjusted B = 0.21, CI 0.08–0.34)	AI/AN ambulatory disability rate between 2014 and 2018 was 9.99% compared to 8.09% among NHW and 7.35% in the total population	NH/PI had greater difficulty walking than NHW (adjusted B = 0.26, CI 0.07–0.44)
	Hypertension^53^	43 (21.9)	64.5% (61.7–67.3) of AA/B with hypertension had uncontrolled disease compared to 51.4% (49.2–53.7) among NHW (p < 0.001)	57.0% (54.3–59.7) of H/L with hypertension had uncontrolled disease compared to 51.4% (49.2–53.7) among NHW (p < 0.001)		
	Diabetes^38,54^	40 (20.3)	Diabetes prevalence among AA/B was 20.4% compared to NHW (p < 0.001)	Diabetes prevalence among H/L was 22.1% compared to NHW (p < 0.001)		
	Infectious Disease^55^	34 (17.3)	AA/B had > 3 times the risk of testing positive for COVID-19 than NHW (RR 3.54, CI 1.38–9.07)	HH/L had > 4 times the risk of testing positive for COVID-19 than NHW (RR 4.68; CI 1.28–17.20		
	Obesity^56,57^	27 (13.8)	AA/B had nearly twice the odds of obesity as NHW (OR 1.89 [1.74–2.05])	H/L had higher odds of obesity than NHW (OR 1.51 [1.43–1.60])	AI/AN had nearly twice the odds of obesity as NHW (OR 1.88 [1.48–2.40])	NH/PI had higher odds of obesity than NHW (OR 1.71 [1.19–2.44])
	HIV^58^	24 (12.6)	Adjusted OR for HIV infection among AA/B compared to NHW is 3.3 (2.6–4.2)			
	Vitamin Deficiency^59^	15 (7.7)	AA/B had nearly 10 times the odds of Vitamin D deficiency compared to NHW (OR 9.6, CI 6.3–14.5)	H/L had 3 times the odds of Vitamin D deficiency compared to NHW (OR 3.2, CI 2.1–4.9)		
	Pain^60^	6 (2.7)	AA/B men had higher acute pain prevalence (14.1%) than NHW men (10.1%), p = 0.004			
Neurological	Stroke^61^	88 (44.9)	Incidence among AA/B was 722 per 100,000 (CI 601–867) compared to NHW (479 per 100,000 [409–561])			
Psychiatric	Depression^62^	83 (42.3)	Average depression symptoms among AA/B were 6.3 (SD 5.32) compared to NHW (5.19, SD 5.32), p < 0.001	Average depression symptoms among H/L were 6.82 (SD: 6.3) compared to NHW (5.19, SD: 5.32), p < 0.001		
	Psychosis^63^	34 (17.4)	AA/B have a higher lifetime prevalence of psychotic symptoms (15.3%) than NHW (9.7%) (p < 0.001)	H/L have a higher lifetime prevalence of psychotic symptoms (13.6%) than NHW (9.7%) (p < 0.001)		
Procedural	Broadband internet access^64^	19 (9.7)	As of 2021, 71% of AA/B had broadband at home compared to 80% of NHW	As of 2021, 65% of H/L had broadband at home compared to 80% of NHW		
	Education levels^65^	19 (9.7)			In 2016, 75% of AI/AN students between ages 18–24 had completed high school compared to 94% of NHW	In 2016, 84% of NH/PI students between ages 18–24 had completed high school compared to 94% of NHW
	Health insurance coverage^66^	17 (8.7)	11% of AA/B were uninsured in 2021 compared to 7% of NHW	19% of H/L were uninsured in 2021 compared to 7% of NHW	21% of AI/AN were uninsured in 2021 compared to 7% of NHW	11% of NH/PI were uninsured in 2021 compared to 7% of NHW

*SE* standard error, *SD* standard deviation, *RR* relative risk, *HR* hazard rate, *OR* odds ratio, *CI* 95% confidence interval.

A majority of examined trials (88%) contained at least one criterion that could disproportionately exclude AA/B, H/L, AI/AN, or NH/PI populations; and 55 trials (28%) had five or more of these criteria affecting at least one of the three populations. As Table 5 demonstrates, the medical category had the majority of criteria that would disproportionately affect diverse populations, including substance use (in 50% of trials, potentially affecting AA/B participants), renal disease (29% of trials, potentially affecting AA/B participants), mobility impairment (27% of trials, potentially affecting every underrepresented group), hypertension (22% of trials, potentially affecting AA/B and H/L participants), and diabetes (14% of trials, potentially affecting AA/B and H/L participants). Within the neurological category, stroke (45% of trials) disproportionately affects AA/B populations, and within the psychiatric category, exclusion criteria include depression (42% of trials) and psychosis (17% of trials) which both disproportionately affect AA/B and H/L populations. The most common procedural criterion that may differentially exclude AA/B, H/L, AI/AN, and NH/PI participants was internet access, found in 10% of trials.

## Discussion

In this content analysis, our objectives were to identify the most common eligibility criteria and determine the prevalence of criteria that may reduce enrollment of underrepresented populations in federally funded AD/ADRD clinical trials. Across all trials, there was an average of 15 eligibility criteria per trial, with Phase I/II pharmacological trials listing the greatest number of criteria per trial. Age exclusions appeared in 87% of trials, making it the number one ranked eligibility criterion identified. The majority of trials had criteria that may act as a barrier to achieving diverse and equitable recruitment into AD/ADRD clinical trials: 82% of trials had poorly operationalized criteria, 88% had at least one criterion that may disproportionately exclude underrepresented populations, and almost 60% of trials did not plan to enroll non-English speaking participants,

The NIH mandates the inclusion of historically underrepresented members of racial and ethnic groups, and persons across the age lifespan, in all NIH-funded clinical research to ensure the generalizability of its clinical research to the entire population^19^. Results of this content analysis indicate that some aspects of the eligibility criteria listed in NIA-funded trials could affect the inclusion of such populations. The high prevalence of poorly operationalized eligibility criteria may increase risk of bias, as they are not well defined and can be subjectively interpreted and applied by the study team. Furthermore, many common criteria analyzed here are disproportionately prevalent among minoritized populations experiencing medical racism and systemic health disparities^20–22^. Language requirements create another potential barrier to diverse enrollment, as English proficiency requirements have been increasing since 2000^23^. If a trial does not provide materials in more than one language, it effectively excludes 7% of Americans who don’t speak English at all and an additional 8% of Americans who speak English less than “very well”^24^. Providing culturally and linguistically appropriate materials, providing interpreters, and hiring bilingual staff could help reduce enrollment disparities and ensure a sample that represents the AD/ADRD population^25^. It is important to note that procedural eligibility criteria (such as transportation, caregiver trial visit attendance, and home internet) may also exclude underrepresented populations. Recent literature on recruitment strategies suggests that establishing diverse study teams, recruiting through multiple venues, precisely defining eligibility criteria, reducing procedural burdens for participants, and using appropriately validated cognitive tests may all help to achieve more balanced representation in AD/ADRD clinical trials^26^.

Age restrictions are another common criterion that may affect generalizability. If age cutoffs are arbitrary, such that there are no clinically relevant differences between an 80-year-old and an 81-year-old not already captured by other eligibility criteria, older adults who may otherwise be willing and able to participate are being differentially excluded. As AD/ADRD is a disease almost exclusively of older adults, excluding older adults from clinical trials for no reason other than age fails to capture the population experiencing the disease in trials of MCI and AD/ADRD participants. Furthermore, as the United States population ages and the projected number of those in the oldest old age groups rises rapidly, it is imperative to recruit and retain older adults from this age group in a broad range of AD/ADRD clinical trials.

Many clinical trials have trouble meeting their deadlines for enrollment, extending trial durations and increasing total cost^27^. A major reason for trial delay and increased cost are protocol amendments, and one of the top reasons for trial amendments is eligibility criteria changes^27,28^. In their 2011 study, Getz et al. found that, of the 6855 protocol amendment changes categorized, 1108 were changes made to the description and eligibility criteria of the trial participants^28^. Protocol amendments to eligibility criteria may also pose a threat to the validity of a trial given that trial populations before and after an amendment may differ when entry criteria are changed during the trial^29^. As such, one avenue to improve the feasibility of a protocol at the onset and save trials time and money may be developing informed processes to develop eligibility criteria.

Our results are aligned with and build upon past literature exploring eligibility criteria for AD/ADRD trials^3,13,25,30,31^. A 2015 review of eight neurological disorder clinical studies, including AD/ADRD studies, found that an average of just six criteria per trial prevented almost three-quarters (73.5%) of screened patients from participating^7^. Rollin-Sillaire et al. found that a high proportion of patients were ineligible for an AD clinical trial due to eligibility criteria that were precautionary but not necessarily needed, including mild vascular abnormalities on MRIs and the use of specific medications to improve cognition^32^. Similarly, Grill and Galvin found that only 10–27% of AD patients are reported as eligible to participate in studies due to eligibility criteria^33^. One challenge to AD/ADRD research is that most of its trials recruit persons over age 65, with a mean age of 73.6 years and older adults are more likely to have comorbid conditions, which are common eligibility criteria in AD/ADRD studies^32,34,35^. Strict eligibility parameters around comorbid conditions is particularly likely to affect AA/B and H/L participants, as these populations tend to have higher rates of dementia-related risk factors such as cardiovascular disease, high blood pressure, and diabetes^36–41^. Furthermore, the number and complexity of diagnostic criteria and tests used to assess cognitive decline in AD/ADRD research can exacerbate participant ineligibility^42,43^. In a recent analysis, Franzen et al. analyzed the eligibility criteria of international Phase II and III MCI and AD pharmacological trials and had similar findings, including that many criteria are poorly defined and that these criteria likely predominantly affect diverse communities^25^.

Addressing concerns regarding eligibility criteria is both necessary and feasible. For example, in 2016, the National Cancer Institute (NCI), FDA, and advocacy groups undertook an effort to identify overly restrictive eligibility criteria in cancer trials and develop guidelines for eligibility criteria to improve accrual and representation^44^. Their process included establishing working groups, reviewing criteria most primed for improvement (such as excluding HIV-positive participants), and assessing improvement in accrual and representation. An impact analysis found that trials that followed NCI’s new eligibility criteria guidelines improved accrual and increased representation of underserved populations^44^. Similarly, the NIA is taking steps to address guidance provided by both FDA and NASEM to help increase the generalizability of its trials’ eligibility criteria. First, all NIA-published clinical trial and clinical trial optional Notice of Funding Opportunities (NOFOs) now require grant applicants to demonstrate that their selected eligibility criteria are representative of the population affected by the disease/condition, generalizable to the wider patient population with this disease/condition, and encourage inclusion of populations outlined in the NIH Inclusion Policies for Research Involving Human Subjects and NIH-designated Populations with Health Disparities^19,45,46^. Second, the NIA recognizes that community input on design can provide additional insights into criteria feasibility before a clinical trial design is finalized and can be obtained through the development of community advisory boards, patient advocacy steering committees, and the inclusion of community health care providers on study research teams^47,48^. NIA is exploring how to further solicit meaningful community input and ensure other lay stakeholders participate in the research process in a systematic and sustainable manner. Third, NIA is tracking progress by implementing Corrective Action Plans (CAPs) to develop effective monitoring and accountability processes. CAPs may address eligibility criteria as an amendment if review determines that criteria are overly restrictive and impeding recruitment goals. Finally, NIA will examine the process used by the oncology field to determine if a similar review of eligibility criteria by the AD/ADRD research community could yield consensus on strategies to broadening eligibility criteria.

Limitations of this analysis include its cross-sectional design based on available data, so changes in patterns of eligibility criteria could not be evaluated. However, investigations that examined the eligibility criteria in AD/ADRD clinical trials funded through other means have reported similar findings^25,49^. Another limitation is that our approach to the literature review of health disparities was maximally conservative and thus is likely an underestimation of criteria that are disproportionately excluding underrepresented participants. Lastly, this study reviewed eligibility criteria set a priori to trial activation and does not analyze real-world recruitment data after a trial has completed participant enrollment. Without access to enrollment data for completed trials, associations between eligibility criteria and enrollment cannot be evaluated. A future study could explore correlational/causal relationships between exclusion categories and actual enrollment to determine the impact of eligibility criteria on actual enrollment of traditionally underrepresented populations.

Strengths of this manuscript include its broad scope. This analysis summarized eligibility criteria across NIA-funded AD/ADRD interventional clinical trials funded over the past five years, representing a substantial number of trials funded since the National Alzheimer’s Project Act was established and providing unique insight across the landscape of recent trials. Additionally, eligibility criteria were captured at a granular level, distinguishing primary from secondary criteria and providing detailed information about which specific conditions are included (instead of capturing criteria solely in broad categories such as ‘medical’). Furthermore, as the need to improve representation in AD/ADRD trials is increasingly recognized in the field, this analysis serves as a starting point to make data-driven determinations about future directions for clinical AD/ADRD research.

Rethinking the rationale behind eligibility criteria using results presented here can assist with meeting recruitment and inclusion goals in a timely manner and reducing trial costs. This work also identifies frameworks and other models that have already been used successfully to improve diverse participant recruitment, which may be adapted to meet the needs of clinical AD/ADRD research.

## Conclusion

Ensuring eligibility criteria balance scientific need with equitable trial design is imperative to adequate representation in AD/ADRD clinical trials. This content analysis provides the first overview of eligibility criteria currently used in U.S. federally funded trials. These findings are an important first step to inform future efforts and identify priorities. Eligibility criteria should have strong scientific justification, diminishing the risk of disproportionately excluding diverse populations and improving external validity of novel treatments and research findings.

### Supplementary Information

Supplementary Tables.

## Data Availability

Data is provided within the manuscript or supplementary information files.

## Abbreviations

AA/B: African American/Black
AD/ADRD: Alzheimer’s disease and related dementias
ADRC: Alzheimer's disease research center
AI/AN: American Indian/Alaska native
CROMS: Clinical research operations and management system
FDA: Food and drug administration
H/L: Hispanic/Latino
MCI: Mild cognitive impairment
NASEM: National academies of sciences, engineering, and medicine
NIA: National institute on aging
NH/PI: Native Hawaiian/Pacific Islander
