# Supplementary material for: Analysis of eligibility criteria in Alzheimer’s and related dementias clinical trials
Source: Sci Rep. 2024 Jul 1;14:15036. doi: 10.1038/s41598-024-65767-x (PMC11217383; doi:10.1038/s41598-024-65767-x)
Supplement: Supplementary file 1 — Supplementary Tables. [file 41598_2024_65767_MOESM1_ESM.docx]

**Supplemental Table 1a: Bonferroni-adjusted ANOVA results for differences in mean number of exclusion criteria by trial type**

|  | **Diagnostic tools, assessments, and imaging studies p-values** | **Non-pharmacological studies p-values** | **Treatment for NPS p-values** |
| --- | --- | --- | --- |
| Non-pharmacological studies p-values | 1.00 |  |  |
| Treatments for neuropsychiatric symptoms p-values | 0.67 | 1.00 |  |
| Pharmacological studies p-values | 0.073 | **<0.001** | **<0.00001** |

**Supplemental Table 1b: Bonferroni-adjusted ANOVA results for differences in mean number of exclusion criteria by target population**

|  | **AD/ADRD p-values** | **At-Risk p-values** | **Cognitively normal p-values** |
| --- | --- | --- | --- |
| At-Risk p-values | 1.00 |  |  |
| Cognitively normal p-values | **0.03** | 0.88 |  |
| MCI p-values | **0.03** | 0.89 | **1.00** |

**Supplemental Table 2a: ANOVA results for differences in mean number of exclusion criteria by trial type**

|  | Degrees of freedom | Sum of Squares | Mean Square Value | F-statistic | P-value |
| --- | --- | --- | --- | --- | --- |
| Trial category | 3 | 6268 | 2089.3 | 24.7 | **<0.001** |
| Residuals | 192 | 16225 | 84.5 |  |  |

**Supplemental Table 2b: ANOVA results for differences in mean number of exclusion criteria by target population**

|  | Degrees of freedom | Sum of Squares | Mean Square Value | F-statistic | P-value |
| --- | --- | --- | --- | --- | --- |
| Target population | 3 | 1264 | 421.4 | 3.8 | **0.01** |
| Residuals | 192 | 21228 | 110.6 |  |  |
